# The Creatine Transporter Gene Paralogous at 16p11.2 Is Expressed in Human Brain

**DOI:** 10.1155/2008/609684

**Published:** 2008-05-19

**Authors:** Nadia Bayou, Ridha M’rad, Ahlem Belhaj, Hussein Daoud, Ramzi Zemni, Sylvain Briault, M. Béchir Helayem, Lamia Ben Jemaa, Habiba Chaabouni

**Affiliations:** ^1^Human Genetics Laboratory, Faculty of Medicine of Tunis, 15 rue Djebel Lakdhar La Rabta, Tunis 1007, Tunisia; ^2^Charles Nicole Hospital, Human Genetics Department, Boulevard 9 Avril, Tunis 1007, Tunisia; ^3^Razi Hospital, Child and Adolescent Psychiatry Department, La Manouba 2010, Tunisia; ^4^Faculté de Médecine de Tours, Institut National de la Santé et de la Recherche Médicale (INSERM) U619, 2bis, Bd Tonnellé 37000 Tours, France

## Abstract

Autism is a complex neurodevelopmental disorder characterized by impairment of social interaction, language, communication, and stereotyped, repetitive behavior. Genetic predisposition to autism has been demonstrated in families and twin studies. About 5–10% of autism cases are associated with chromosomal abnormalities or monogenic disorders. The identification of genes involved in the origin of autism is expected to increase our understanding of the pathogenesis. We report on the clinical, cytogenetic, and molecular findings in a boy with autism carrying a de novo translocation t(7;16)(p22.1;p11.2). The chromosome 16 breakpoint disrupts the paralogous SLC6A8 gene also called SLC6A10 or CT2. Predicted translation of exons and RT-PCR analysis reveal specific expression of the creatine transporter paralogous in testis and brain. Several studies reported on the role of X-linked creatine transporter mutations in individuals with mental retardation, with or without autism. The existence of disruption in SLC6A8 paralogous gene associated with idiopathic autism suggests that this gene may be involved in the autistic phenotype in our patient.

## 1. Introduction

Autism is defined as a pervasive neurodevelopmental disorder with onset before 36 months of age, resulting in impairment in social interaction, communication, and in a manifestation of repetitive stereotypic behavior. Its incidence is estimated at about 1/1000 to 1/2000 with a biased male-to-female ratio of three or four to one (3-4:1) [1]. The exact aetiology of autism remains unknown, although it is likely to result from a complex combination of genetic, neurological, immunological, and environmental factors [2]. Genetic factors for autism have been well established in family and twin studies. The concordance rate in monozygotic twins is estimated to be 90%. This rate is between 0% and 24% in dizygotic twins [3].

About 3–6% of autism cases are associated with chromosomal abnormalities [4]. Positional cloning of translocation or inversion breakpoints has been a successful way of identifying candidate genes for many genetic disorders. The underlying assumption is that a gene disrupted by the breakpoint is causative of the phenotype. Therefore, as an alternative way to identify candidate genes for autism, we analyzed the breakpoint regions of de novo translocation t(7;16)(p22.1; p11.2) in a patient with autism. To the best of our knowledge, there are few published reports of patients with autism carrying chromosomal translocation located on 16p11.2 [5].

## 2. Materials and Methods

### 2.1. Case report

Our patient is the younger of two children born at term of non consanguineous healthy parents. Patient's psychomotor delays were noticed in the first months of life. He was unable to maintain eye contact and presented many motor stereotypes and ritualistic behaviors such as cutting papers into small pieces and aligning objects. Pregnancy was uneventful. Delivery was by caesarean section secondary to a narrow basin. Birth weight was 3350 g, and no recognized malformations were noted. The patient could not sit alone before 12 months neither could he walk alone before 26 months. He never achieved continence and still needed help in eating, and complete assistance in dressing and self care.

The patient had severe language impairment being only able to pronounce monosyllabic words, without any relational intent. A clinical evaluation performed at the age of 4 years revealed a weight of 13 kg (−2SD), a height of 102 cm (normal), and an occipitofrontal head circumference of 48 cm (−2, 3SD). No evident dysmorphic features were noted.

The clinical diagnosis of autism was made according to the Diagnostic and Statistical Manual of Mental Disorders—Revision 4 (DSM-IV) and the Autism Diagnostic Interview, French version 6 1993 (ADI-R) criteria at the age of 4 years and 11 months. The Childhood Autism Rating Scale, French version (CARS-T) scale indicated a score of 45 documenting a high level of autistic behavior. The IQ test was crucial in confirming the mental retardation diagnosis. It was not carried out as the parents declined this. The MRI of the brain revealed a cerebellar megacisterna in the posterior fossa. The MRS showed a normal spectral pattern as well for the creatine as for the remaining metabolites. Creatine and creatinine concentrations in body fluids were not evaluated as the parents refused this. Southern blot analysis revealed the absence of FRAXA mutation. The healthy sister was not available for analysis.

### 2.2. Cytogenetic and FISH studies

Chromosome analysis was performed on the proband's and parent's blood, using standard high-resolution techniques. FISH was performed on the proband's metaphase with BACs that were selected according to the UCSC Genome Browser, and provided by BACPAC Resource Centre (BPRC) and Pr. Mariano Rocchi (University of Bari, Italy). BAC clones were biotinylated with biotin-11-dUTP (Sigma, Mo, USA) by nick translation using the BioNick labeling system (Invitrogen Life Technologies).

### 2.3. Bioinformatic research

Bioinformatic search was carried out using the UCSC Genome Browser to identify potential autism candidate genes. The Genomatix Software was used for the promoter and the gene structure predictions.

### 2.4. Expression studies

Total RNA was isolated and purified using the RNAgents Total RNA Isolation System (Promega) from peripheral white blood cells and other human tissues (testis, adult and foetal brain, fibroblasts, and kidney). 1 *μ*g of total RNA was converted to cDNA was used in 25 *μ*L reactions with AMV PCR Master Mix (Promega).

A 4 nucleotides insertion in the 1st exon of the SLC6A8 paralogous gene let us to design specific primers with Web Primer DNA program (insertion not found in the SLC6A8 gene).

RT-PCR assays specific for the chromosome 16 paralogous SLC6A8 transcript and direct sequencing were done using these specific primers (5′ -TACCGCTTCTTCTCGCGGCTCTTG-3′ , and 5′ -ATCGCGCGCGGCGGCACGGC-3′).

## 3. Results

**Figure 1:**
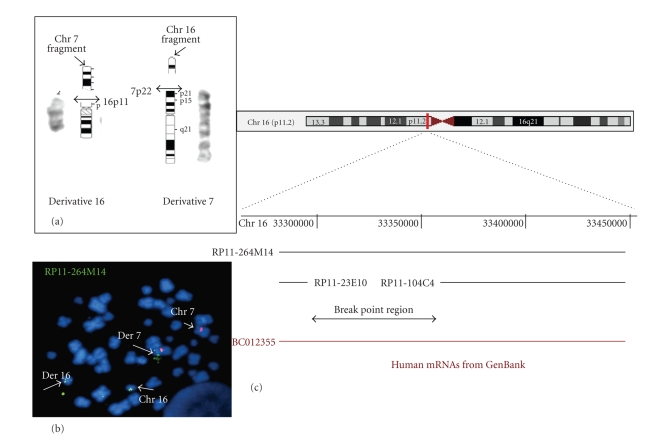
Physical mapping of the breakpoint on chromosome 16. (a) The patient derivative chromosomes 7 and 16 are shown. By comparison to the both respective ideogrammed chromosomes, the breakpoints were located in 7p22 and 16p11.2. (b) FISH analysis with the BAC RP11-264M14B22 (green) located in 16p11.2 and a centromeric probe of chromosome 7 (red) shows that this BAC is spanning the breakpoint on chromosome 7. (c) Physical map of genomic region 16p11.2 derived from the May 2006 version of the UCSC Genome Browser (http://www.genome.ucsc.edu/). The genomic clones covering the region flanking the translocation breakpoint are indicated by simple black lines. The arrow indicates the position of the translocation breakpoint with regard to the genomic clones. The red line represents the location of the predicted gene.

The G-band pattern established a 46, XY, t(7;16)(p22; p11) karyotype (Figure 1(a)). The mapping of the chromosome 7p22.1 breakpoint demonstrates that no gene was disrupted in the interval (data not shown).

As shown in Figure 1(c), the RP11-264M14 BAC hybridized both to derivative 7 and derivative 16 and to the normal chromosome 16 but not to the normal chromosome 7 (Figure 1(b)), indicating that this clone spans the translocation breakpoint on chromosome 16p11.2 (Figure 1(c)).

The clone RP11-264M14, on 16p11.2, was found to be devoid of known genes but it included an entire mRNA predicted to be paralogous to the human creatine transporter gene (SLC6A8) located on Xq28. The chromosome 16 paralogous gene is spanning 486 952 bp of chromosome 16 (33,205,876-33,692,827).

Our bioinformatic research ruled out with the Genomatix Software predicted for the paralogous gene a structure of 13 exons with intron-exon junctions conforming to splice-site consensus sequences (Table 1).

The predicted mRNA for this paralogous gene (XR_017514) spans 3300 bp with a coding sequence of 1326 bp and an ORF of 441 amino acids. A predicted 1414 bp promoter is located between nucleotides 33, 684, 872, and 33, 686, 285 of the genomic DNA sequence contig NT_010393. The cDNA sequence contained 1846 bp of 5′ -UTR and 1230 bp of 3′ -UTR.

NCBI BLAST homology search analysis revealed that the nucleotide sequence of this paralogous gene is 97.1% similar to the SLC6A8 gene.

**Figure 2:**
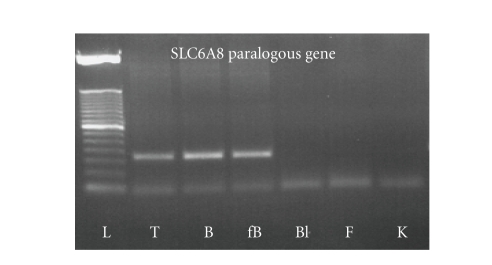
Expression pattern of the SLC6A8 paralogous gene. (RT-PCR was done on RNA extracted from various human tissues. L: ladder; T: testis; B: brain; fB: foetal brain; Bl: blood; F: fibroblast; K: kidney).

RT-PCR assays specific for the chromosome 16 paralogous SLC6A8 transcript and direct sequencing by primers identified a specific expression in foetal and adult brain and in testis. The results indicated no expression of this gene control blood cells and fibroblasts (Figure 2). Although we did not identify the actual sequence of the breakpoint, we mapped it between 33.28 Mb and 33.31 Mb corresponding to the promoter region.

## 4. Discussion

Substantial evidence suggests that chromosomal abnormalities contribute to autism risk but the exact prevalence is unclear because literature surveys span different diagnostic and cytogenetic approaches and sample sizes. Recent studies show rates of detected chromosomal abnormalities in 5%–10% of affected individuals [6].

A genome-wide analysis of structural variations ruled by Marshall et al. [7] identified an autism susceptibility locus on 16p11.2 involving deletions and duplications. They reported that this copy number variation (CNV) region was found at near to 1% in the studied autistic cohort and not in controls.

A region on chromosome 16p11.2 (from genomic coordinates 29.5 Mb to 30.1 Mb) was found deleted de novo in autistic patients [8]. Kumar et al. [9] suggested that the 16p11.2 microdeletion is one of the most common recurrent genomic disorders associated with autism. The question to rise is whether these deletions removed the coding regions of some genes. This region is nearby of the translocation breakpoint characterized in our autistic patient (from genomic coordinates 33.28 Mb to 33.31 Mb) corresponding to the promoter region of the human creatine transporter gene (SLC6A8) paralogous.

This gene has been localized to 16p11.2 by two studies [10, 11]. It is also known as SLC6A10 or CT2 [12]. The mRNA sequence BC012355 reported by Strausberg et al. [13] is 2168 bp with a coding sequence of 1908 bp and 636 amino acids predicted.

Eichler et al. [11] and Höglund et al. [14] have identified the SLC6A10 gene as a pseudogene with an early stop codon. However, this gene has been considered to be a functional gene both in UniGene at NCBI as well as in a review article by Chen et al. [15].

This gene was found specifically expressed in testis by Iyer et al. [10]. They suggested that this gene function is critical for creatine transport into the cell to ensure normal sperm mobility.

Our data extend this finding; this is the first time this phenotype has been reported for the chromosome 16 SLC6A8 paralogous, and the first time expression of this gene has been reported in brain as well as testis.

The SLC6A8 genes are members of a superfamily of proteins that includes the family of Na^+^ and Cl^−^ dependent transporters responsible for the uptake of certain neurotransmitters (e.g., dopamine, GABA, serotonin, and noradrenaline) and amino acids (e.g., glycine, proline, and taurine) [16].

Szatmari et al. [6] reported that some members of the solute carrier family fall in positive linkage regions for autism or close to linkage peaks. Even if they have not glutamatergic synaptic function, some members affect brain development and constitute excellent candidate genes for autism.

There is a high prevalence of SLC6A8 mutations in X-linked mental retardation. Common features of X-linked mental retardation are neurological disturbances including seizures, behavioral problems, speech delay, and inability to engage in structured play [17]. Our patient was diagnosed with a high level of autistic behavior and severe delay both in speech and in expressive language function. We hypothesized that this phenotype is the result of the creatine transporter paralogous disruption by the translocation breakpoint on 16p11.2.

The disruption of this gene may cause a decreased dosage responsible for specific tissue and developmental effects leading to autistic traits.

This data is in agreement with the deletions on 16p11.2 reported by Marshall et al. and carried only by autistic patients [7]. The authors suggested that the loss of some genes in the interval is critical for the development of speech, language, and communication.

As gene expression is the basis of many crucial functions in the cell, the hypothesis is that perturbation in the network of genes by the translocation or the CNVs in this specific region cause the related pathology by dosage differences and underlie the autism phenotype. The absence of expression of the predicted human creatine transporter paralogous gene (SLC6A10) in accessible tissues makes it currently impossible to investigate this further.

Confirmation of whether a defective chromosome 16 paralogous SLC6A8 gene causes autism should come from observing rearrangements of this gene in other autistic patients. Coding and intronic regions have to be screened to establish if mutations in the SLC6A8 paralogous gene are the cause of autism in a fraction of nontranslocation subjects. Validation of the candidate gene identified here will rely on association studies in patients and families with autism.

## Figures and Tables

**Table 1:** Exon/intron organization of the human SLC6A8 paralogous gene.

Exon no.	Exon size	Sequence at exon/intron boundaries	
	(bp)	5′ donor	3′ acceptor
1	266	NA	CGGAGgtgag
2	132	cccagGTGTG	CAAAGgtgag
3	250	cccagGCCTG	TGGGAgtgag
4	133	cctagGAACA	GAAAGgtacc
5	135	cccagATCGT	CTCAGgtgag
6	103	tctagGTATG	TACAAgtaag
7	125	cccagCAGCC	GTCAGgtagg
8	113	cacagGGCGG	GCTTGgtctc
9	138	cacagTTTGT	CTGATgtgag
10	103	cccagGGTGG	GTATGgtagg
11	101	cacagGAGCT	GCATGgtaag
12	171	tgtagGGCAT	CTGAGgtaag
13	1984	tgcagTGCTG	NA

*Intron nucleotides are in lowercase letters, exon nucleotides are in capital letters.

**NA: not applicable.
